# Use of a public park for physical activity in the Caribbean: evidence from a mixed methods study in Jamaica

**DOI:** 10.1186/s12889-019-7247-6

**Published:** 2019-07-08

**Authors:** Colette A. Cunningham-Myrie, Tamika Y. N. Royal-Thomas, Althea E. Bailey, Jeanette Gustat, Katherine P. Theall, Joy E. Harrison, Marvin E. Reid

**Affiliations:** 1 Department of Community Health and Psychiatry, University of the West Indies, 3 Gibraltar Camp Way 7, Mona, Jamaica; 2 Tropical Metabolism Research Unit, Caribbean Institute for Health Research, University of the West Indies, Mona, Jamaica; 3 Mathematics & Statistics Department, The College of New Jersey, Ewing, NJ USA; 4 Department of Epidemiology School of Public Health and Tropical Medicine, Tulane University, New Orleans, Louisiana USA; 5 Department of Global Community Health and Behavioral Sciences School of Public Health and Tropical Medicine, Tulane University, New Orleans, Louisiana USA

**Keywords:** Physical activity, Parks, Built environment, Direct observation, SOPARC, Mixed methods, Developing country, Caribbean

## Abstract

**Background:**

Small island Caribbean countries such as Jamaica are now facing an epidemic of obesity and decreased physical activity (PA) levels. Public parks have been shown to be important resources for PA that also provide psychological and social benefits associated with increased PA. There are no studies that document PA in parks in the Caribbean.

**Methods:**

This study utilized a mixed method approach by using the System for Observing Play and Recreation in Communities (SOPARC) to obtain baseline data on park usage patterns in Emancipation Park, a large urban public park in Jamaica. In addition, in-depth interviews were conducted to gain additional insights on the park’s use for PA.

**Results:**

The park was used mostly by females, in the evenings and by persons 18–64 years old. Females had significantly lower mean energy expenditure (EE) than males (0.078 versus 0.080 kcal/kg/min, *p* < 0.05). In-depth interviews revealed that safety, a central location within a business district, aesthetic appeal, a walking track and individual health benefits were key reasons for persons engaging in PA at the park.

**Conclusions:**

This is the first study to describe the usage of a public park for PA in Jamaica. The study elicited aspects of park use for PA in a major urban park in Jamaica from different vantage points by using direct systematic observation augmented with a qualitative approach. It revealed important differential park use for PA by sex, age group and EE levels, and provided insights into factors that motivate and hinder park usage for PA. This can be used by policymakers in Jamaica to inform PA interventions to reduce obesity, provide baseline data for comparisons with other parks in developing countries and to advocate for well-designed public parks.

**Electronic supplementary material:**

The online version of this article (10.1186/s12889-019-7247-6) contains supplementary material, which is available to authorized users.

## Background

Chronic Non- Communicable Diseases (NCDs) are now at epidemic proportions in Jamaica, a small island developing country within the Caribbean, accounting for over 5% of Gross Domestic Product [1] and are the leading causes of death [2]. Nationally representative data from the two Jamaica Health and Lifestyle Surveys (JHLSs) completed in 2000 (JHLS I) and 2008 (JHLS II) respectively among 15–74-year-old persons, documented increased prevalence of obesity (19.7% versus 25.3%) [3]. Another study, the Jamaica Youth Risk and Resiliency Survey (JYRRS) revealed that greater than 20% of 15–19-year-old adolescents were overweight/obese [4].

Physical activity (PA) is a modifiable risk factor for overweight/obesity. The JHLS II revealed increased prevalence for low PA levels (from 36 to 46%), inclusive of significant widening of the sexual dimorphism (female: 62% versus 41%; male: 28% versus 21%) [3]. Jamaican policymakers have engaged in several initiatives to curtail this risk factor. For example, in September 2007, Jamaica was a signatory to the ground-breaking Caribbean Community (CARICOM) Port of Spain Declaration, emanating out of a Regional summit on NCDs; this included a commitment to develop the physical and social environment to promote physical activity by providing areas which are easily accessible, safe and well maintained [5]. The Jamaican government also developed a 5-year national chronic disease strategic plan covering the period 2013–18, with one of the specific objectives targeting the reduction of the proportion of persons engaging in insufficient PA by 5% by 2018 [6]. Most recently a social marketing campaign, dubbed ‘Jamaica Moves’ has been implemented to increase PALs and raise the awareness of the link between PA and chronic diseases [7].

Public parks have been shown to be important resources for recreational PA [8–11], and usage patterns correlate with proximity, condition and types of facilities [10, 12–15]. They also provide psychological and social benefits that are associated with increased PA [16, 17]. Jamaica, a small island developing country, has public parks of varying sizes and conditions in many communities but although free or relatively inexpensive to use, most remain largely underutilized for PA. In fact, secondary analysis of the JHLS II additionally found that neighborhood recreational space availability was counterintuitively associated with low levels of PA among Jamaican females, the authors opining that the type, quality and safety concerns were possible influencing factors [18].

To our knowledge there are no scientific studies that have documented park usage patterns in Jamaica or the other English-speaking islands in the Caribbean region. The specific park for our research, is the Emancipation Park. It is unique, in that it is the only large urban public park where unusually large numbers and mixture of persons of both sexes, across age groups and from many different backgrounds on a daily basis use various spaces for all levels of PA and not just organized sport. This study uses a mixed methods approach which allowed us to describe patterns of use of the park by sex, age group category and areas within the park and also to better understand and explore the reasons for the positive deviance of persons who use a public park for PA, given the high levels of physical inactivity among Jamaicans [3].

We hypothesized that PA levels and energy expenditure (EE) in Emancipation Park would differ by sex, age group category and areas within the park based on its design and features. Assessing the usage patterns will provide novel baseline data on the demographic profiles of users, types of PA, levels of EE for small-island countries within the Caribbean region and the findings should prove useful for understanding barriers and facilitators to increasing PA in public parks and monitoring and evaluating investments of public resources into the use of public parks for PA by policymakers. The aims of this study were to a) obtain baseline data on current usage patterns and EE in Emancipation Park and b) gain additional insights into how and why persons use Emancipation Park for PA. As far as we are aware, this represents the first scientific examination of this context in Jamaica and the English-speaking Caribbean.

## Methods

### Study setting

**Fig. 1:**
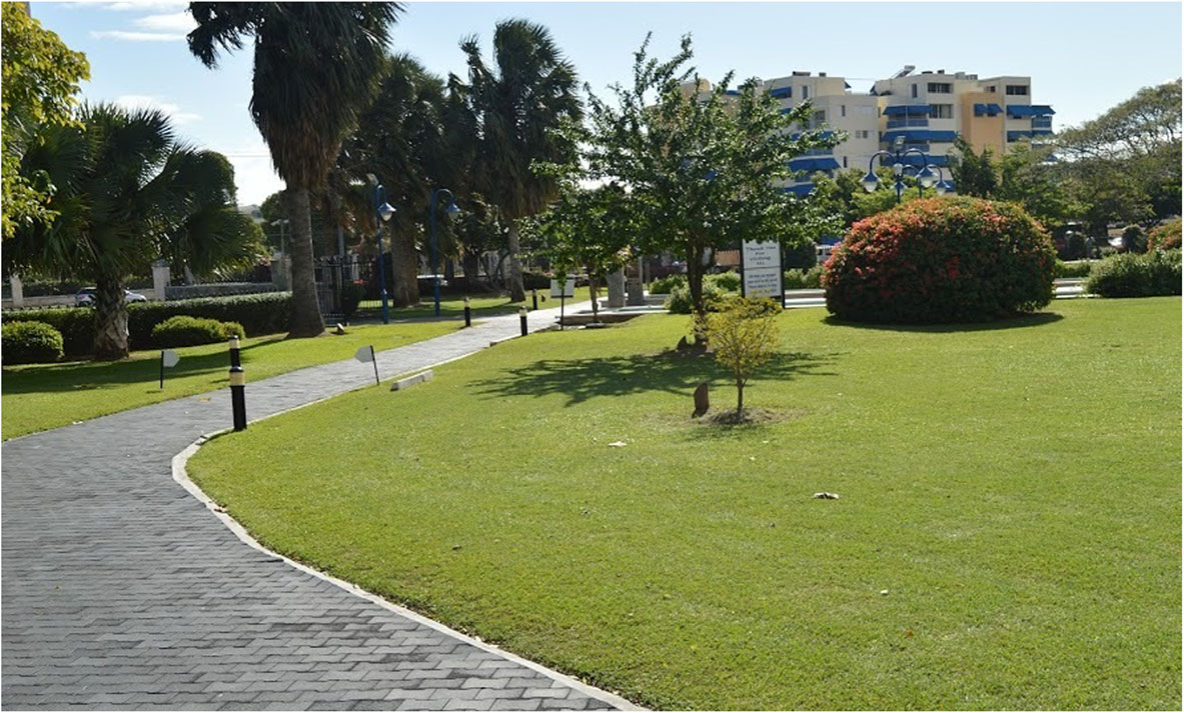
Circumferential track at Emancipation Park, Jamaica

**Fig. 2:**
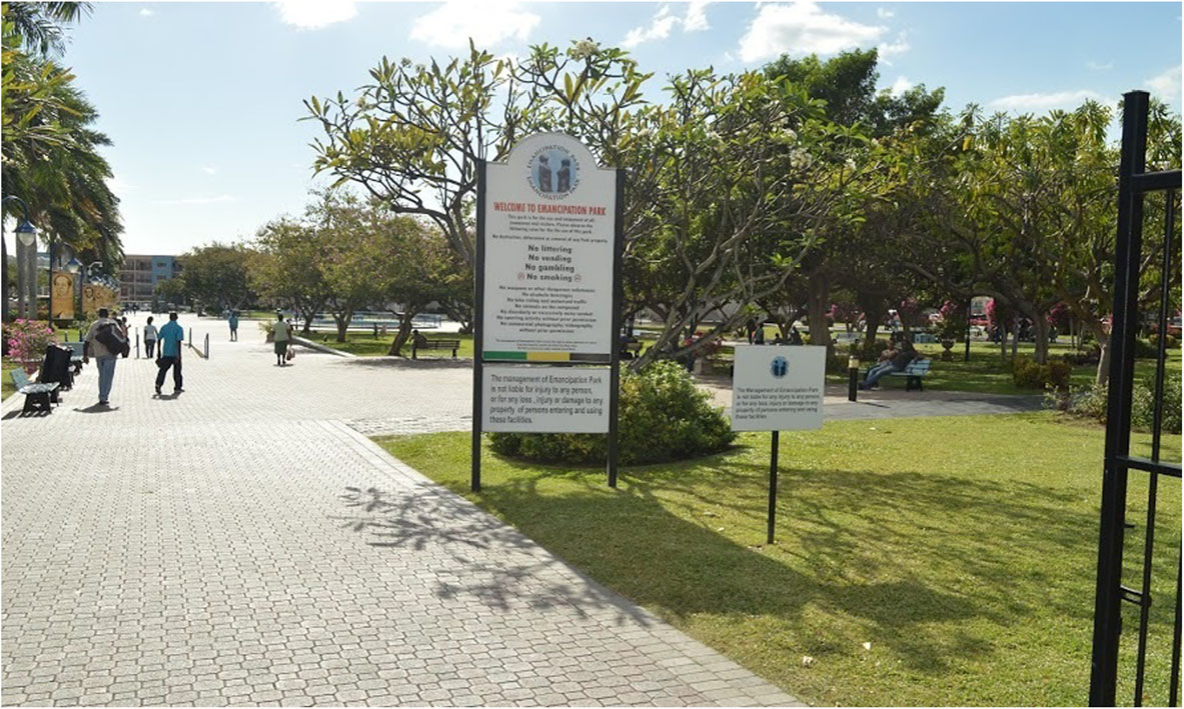
Main walkway from eastern entrance of Emancipation Park, Jamaica

**Fig. 3:**
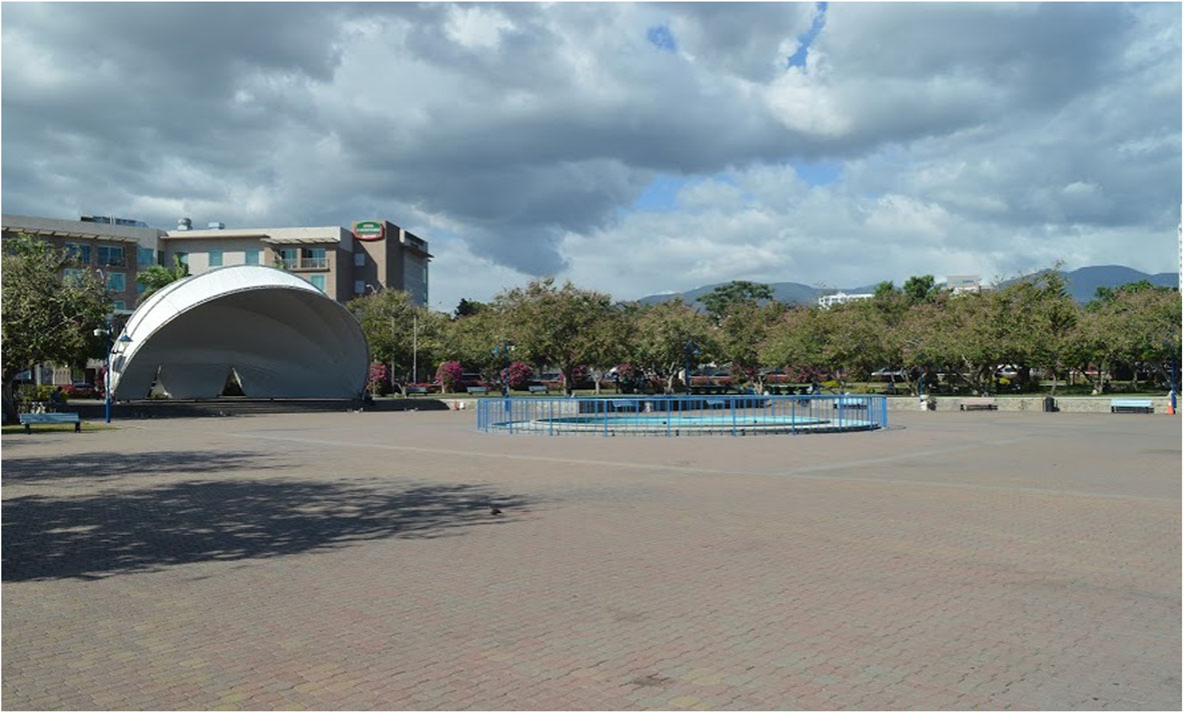
Promenade surrounding bandstand at Emancipation Park, Jamaica

Emancipation Park is a public park officially opened in 2002 and managed by the government through the National Housing Trust, a statutory body [19]. The well-maintained park occupies approximately 7 acres (28, 328 m^2^), in the urban business district of New Kingston in the parish of St. Andrew, Jamaica. Previously a large dustbowl, the park was transformed into an oasis using public funding to include a circular 500 m track paved with unitary surface (see Fig. 1), many attractively landscaped green areas and large concrete promenades with small pools of water and a large fountain. There is a main walkway running from east to west of the park (see Fig. 2) and a large concrete bandstand with surrounding promenade is a focal point for concerts and group exercise classes (see Fig. 3). The park has complete perimeter fencing, security guards and pedestrian access is through 3 main gates.

There is no other similar public park in Jamaica with all these aforementioned features in one location, and as stated previously, large numbers of persons from many different backgrounds use the park for all levels of PA.

### Data collection

This study focused on persons using Emancipation Park. Systematic observations of PA and semi-structured intercept interviews were conducted in the park. Field notes from interviews were also recorded.

### Quantitative assessment using systematic observation

The System for Observing Play and Recreation in Communities (SOPARC) [20] was employed in the study to assess usage and PA levels. SOPARC incorporates momentary time sampling techniques that are both systematic and periodic to gain objective observational data on contextual and individual physical activity, including the estimation of energy expenditure [21]. The original SOPARC coding forms used by McKenzie et al. were slightly modified by removing the racial categories and using only three age group categories. SOPARC has been utilized in a number of developed and developing countries such as the USA [8, 22], Australia [23], Belgium [24] and Brazil [25]. Prior to beginning the study, research staff were trained in SOPARC protocols, during multiple classroom type workshops and field visits to various public parks, including Emancipation Park. Trainees were taught to conduct momentary systematic scans from left to right in clearly identified target areas (TAs) and interobserver agreement tested to ensure at least 80% agreement. TAs are mutually exclusive, arbitrary subdivisions of the park predetermined by the study team for ease of observation. Twenty seven TAs were defined and mapped within Emancipation Park. The TAs primarily included green spaces such as grassy areas for unspecified activities, open paved areas for congregating around the bandstand, and paved areas along constructed water features. Bathroom facilities and park offices were along one of the TAs. Several of the TAs included benches and a few contained fixed table tennis and tables marked for checkers or chess games that were made of concrete. Additionally, the circular track was assessed as a separate area. Following SOPARC protocols, each TA was assessed for the following: a) contextual data on the following conditions of the target areas: accessibility and usability, presence of supervision and equipment presence, degree of lighting, classification of organized activities and b) the demographic features of sex and age categorized as < 18 years, 18–64 years and ≥ 65 years and c) level of PA coded as sedentary (lying down, sitting, standing), walking or vigorous activity (for example walking briskly, walking with weights, jogging, running, aerobics). Separate scans were done for each sex, firstly females and then males.

Scans of each TA were conducted four days per week, four times per day (6–7 am; 12:30–1:30 pm; 2:30–3:30 pm; 5:30–6:30 pm) over the four-week period from June 16–July 12, 2015. The path was assessed separately. Observers were stationed at one point and counted users for 42 s during each observation time (the approximate time of walking one lap) [20]. This frequency was based on the recommendations for obtaining a robust estimate of park user characteristics and PA using SOPARC by Cohen at al [26]. If all TAs were scanned in less than an hour then scans were repeated sequentially, starting with the lowest numbered target area until the 1-h observation period had elapsed. Repeat observations were collected daily for 13 randomly selected predetermined 1-h observation periods (20% of all observations) by a pair of trained assessors who simultaneously and independently conducted observations.

### Qualitative assessment using in-depth interviews

In-depth interviews used a concurrent nested approach to explore and understand user perceptions of Emancipation Park regarding PA and their use of the space. The use of qualitative strategies for in-depth exploration of phenomenon is described by Creswell (2009) *“as a means for exploring and understanding the meaning individuals or a group ascribe to a social or human problem”* [27]. Semi- structured interviews, aided by the use of an in-depth interview topic guide (Additional file 1), were used to collect detailed qualitative data for this study.

Participants were purposively selected for interviews based on sex, PA level observed and age group categorization. Participants were approached after the interviewer had observed the individual in the Park engaged in one of three PA levels (sedentary, walking or vigorous). Individual persons engaged in walking or vigorous PA were approached after completion of the activity.

Five in-depth semi-structured interviews were done between June and September 2015. Interview questions focused on gaining insights into the use of and perceptions about Emancipation Park for PA. Questions addressed included why they visited Emancipation Park, how the Park helped them to decide to be active, barriers to using the park and how to encourage others to use the park for PA. The interviews lasted between 10 to 30 min and were conducted at times separate from the observation periods for the SOPARC.

### Data analysis

#### Quantitative analysis

Descriptive statistics were used to describe the park user characteristics. Counts for each TA for each scan were summed. The proportion of scans where at least one person was observed during the observation period was examined. Counts were averaged when a target area was scanned more than once in the scheduled scan period. The proportion of individuals engaged in sedentary, as well as moderate to vigorous PA (MVPA), such as walking, running, aerobics, etc. was examined. Chi-square tests were used to examine differences between age and sex groups by four target areas (based on anticipated highest use for PA and highest levels of MVPA), using chi-square goodness of fit tests to compare proportions with more than two categories and two sample proportion z tests where there were only two categories. Chi-square tests for trends were also conducted. T-tests for two independent groups were done to compare the mean energy expenditure (EE) between males and females by target area types.

The units of observation were the counts observed (observations). Park user characteristics assessed also included: presence of any park users – proportion of scans where at least one person was observed during the observation period; number of park users – count of number of park users within scan area where at least one person was observed. Kappa statistics were used to assess interobserver agreement on contextual data; Pearson correlation coefficient (r) and intraclass correlations for count data for paired observations were also calculated.

Energy expenditure (EE) rates were calculated for the 4 target area types as well as for each sex according to previously validated physical activity codes [28]. The number of persons observed engaged in each EE category was multiplied by the respective EE value (sedentary 0.051 kcal/kg/min, walking 0.096 kcal/kg/min or vigorous 0.144 kcal/kg/min). The EE for each PA category was summed to obtain the total EE for each sex and target area. The mean EE was computed by dividing the total EE by the number of persons observed for each sex and target area. Analysis of Variance (ANOVA) was conducted to examine the variance among the mean EE score for each target area for the total sample and this was also done within each sex category.

All analyses were done using Stata version 12.1 and findings deemed statistically significant at *p* < 0.05.

### Qualitative analysis

The interviews were recorded and transcribed verbatim. These transcripts as well as the field notes from the 20 min semi-structured observation and the researcher’s reflective memos were analyzed by two independent researchers using constant comparative analysis [29, 30]. The data were read several times to ensure familiarity. Open coding i.e. “the labeling of concepts, defining and developing categories based on their properties and dimensions” [31] was used to create the initial coding framework and to identify themes within the data. The researchers independently reviewed the initial coding framework for duplications to develop a shorter list of categories and cross referenced each other’s work after manual coding. Discrepancies were discussed until consensus was reached.

The quantitative results derived from application of the SOPARC methodology were compared with the qualitative findings to reduce codes and categories and to form overarching themes derived from participants’ experiences and perceptions.

## Results

### Quantitative findings

#### Reliability

Data from a total of 429 simultaneous scans were used in the reliability analysis. Inter-observer agreement (IOA) scores (not shown) for contextual variables were perfect for area accessibility and above 99% for usability, degree of lighting and presence of organized activity; the sex-specific IOA scores for age grouping were 72.8% with slight differences by activity levels (70.4% for females; 67.9% for males). All coefficients met acceptable criteria for reliability assessment ranging from r = 0.97 to 1.0 (data not shown). Specifically, reliability was high for all counts across total users [Interclass correlation coefficient (ICC) 95% CI: 99.75–99.95)]; sex (ICC 95% CI: 99.94–99.99), age group (ICC 95% CI: 96.78–99.96), and activity level (ICC 95% CI: 93.02–99.90).

#### Park user characteristics

A total of 9,915 persons were observed during 2141 separate scans. Each researcher counted an average of 191 visitors per hour. Significantly more females than males used the park (females = 52, 95% CI = 51, to 53%; males = 48, 95% CI = 47 to 49%, *p* < 0.001) and most users (74%) were adults 18–64 years old. Of all users, 51.4% were observed engaged in sedentary activity viz. sitting, standing or lying down compared with 36.5% walking (*p* < 0.001). Overall, the proportion of persons engaged in MVPA was 46.8%, with 99.9% of users of the walking track engaged in MVPA.

#### Sex-specific park use

Table 1 illustrates the sex-specific park user characteristics according to age group and PA level. There was significantly higher use of the park by both sexes in the evening period (females =58.3% versus males =49.4%, *p* < 0.001). When usage patterns were examined by age group, over 70% of users were between 18 and 64 years old.

**Table 1:** Sex-specific characteristics of park users by age group and physical activity level

	Total Female Activity (*n* = 5179); Total Female Age group (*n* = 5162)						Total Male Activity (*n* = 4766); Total Male Age group (*n* = 4753)					
	Activity Level (%)			Age group (%)			Activity Level (%)			Age group (%)		
Variable	S	W	V	< 18	18–64	≥65	S	W	V	< 18	18–64	≥65
Observation Period												
Morning	1.41	12.22	2.99	0.17	12.03	4.15	3.59	10.62	4.32	0.53	12.58	5.45
Lunch	6.35	3.77	0.83	2.58	8.10	0.33	8.41	4.39	0.97	2.86	9.00	1.85
Afternoon	9.65	4.11	0.58	3.35	10.52	0.48	12.61	4.62	1.22	3.26	12.29	2.78
Evening	34.31	17.86	5.91	9.78	47.09	1.41	26.37	15.25	7.64	9.66	35.96	3.79
Day of Week												
Monday	6.01	5.04	1.33	0.74	10.87	0.81	5.60	4.45	1.95	0.80	8.96	2.21
Tuesday	5.37	4.61	2.09	0.79	10.44	0.87	5.64	3.08	1.66	1.35	7.41	1.64
Wednesday	8.88	8.38	1.87	2.25	15.48	1.16	9.00	7.15	3.23	2.55	14.77	2.19
Thursday	7.34	8.59	2.51	3.62	13.29	1.47	8.83	8.16	3.25	3.30	13.76	3.11
Friday	3.86	2.53	0.37	1.41	4.80	0.66	3.61	1.64	0.44	1.01	3.93	0.78
Saturday	13.13	6.01	1.26	4.30	15.21	0.97	11.75	7.07	2.39	4.36	14.03	2.78
Sunday	7.14	2.80	0.89	2.77	7.65	0.43	6.55	3.32	1.22	2.95	6.96	1.16
Total (%)	51.73	37.96	10.31	15.89	77.74	6.37	50.99	34.87	14.14	16.31	69.83	13.86

*S* Sedentary, *W* Walking, *V* Vigorous

The percentage for female’s activity levels was done out of the total for female’s activity. The percentage for male’s activity levels was done out of the total for male’s activity. The percentage for female’s age group was done out of the total for female’s age group. The percentage for male age group was done out of the total for male’s age group

Most of the persons observed using the park were sedentary with just over half (51.7%) of all females (*p* < 0.001) and 51.0% of all males (*p* < 0.001) falling in this category. When the PA level of each sex was examined by period of the day, the highest proportions for both sexes engaged in vigorous-intensity activity levels were in the evening period (females = 57.3%, males = 54.0%, *p* < 0.001).

**Fig. 4:**
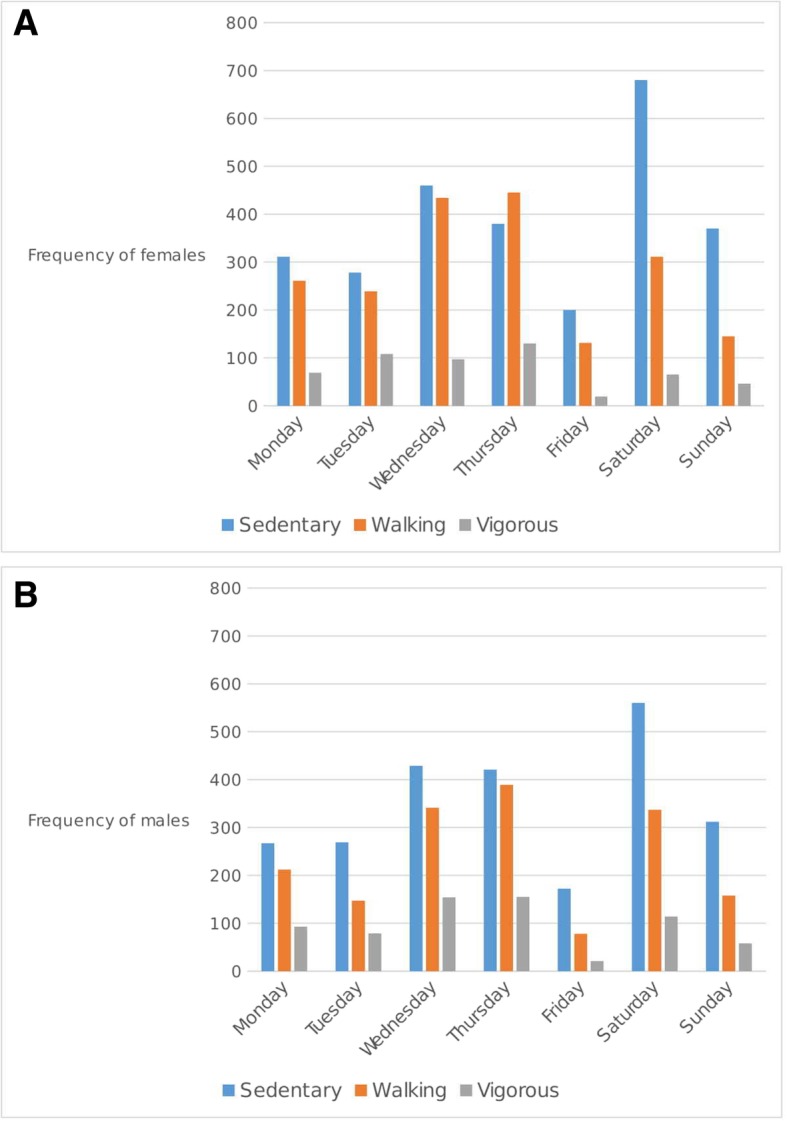
Daily Physical Activity Levels in Emancipation Park for females (Panel A) and males (Panel B)

When usage patterns were examined by weekday, among females a minority of seniors (persons ≥65 years old) used the park on all days of the week except on Monday when the < 18-year olds had the lowest frequency (p < 0.001). Among males, the 18–64-year-old group visited the park at significantly higher levels than the other age groups irrespective of the observation day (p < 0.001). User counts within age groups revealed statistically significant differences according to the day of the week for any of the age-groups (p < 0.001). Fig. 4 illustrates that for most days, both sexes were engaged in significantly more sedentary activity compared with walking and vigorous-intensity PA level (p < 0.001).

Table 2 shows the Chi-squared tests results on the counts of persons within the target area types and sex-specific age group categories. There were significantly more adults (18–64 years) versus the other age groups within the target areas, (χ^2^ = 724.49, *p* < 0.0001). Significantly more adult females (χ^2^ = 503.56, p < 0.0001) and adult males (χ^2^ = 312.60, p < 0.0001) were also seen within the target areas. The walking track was least used by the young (6.3%), while 22.6% of adults and 44.4% of seniors did. On the other hand, the green spaces were mostly used by the youth (59.5%), compared to 39.4% of adults and 21.0% of seniors.

**Table 2:** Sex-specific use of target areas by age group

Target Area	Total χ^2^ = 724.49, p < 0.0001			Females χ^2^ = 503.56, p < 0.0001			Males χ^2^ = 312.60, p < 0.0001		
	< 18 *(%)*	18–64 *(%)*	> 64 *(%)*	< 18 *(%)*	18–64 *(%)*	> 64 *(%)*	< 18 *(%)*	18–64 *(%)*	> 64 *(%)*
Walking track	101 *(6.3)*	1656 *(22.6)*	439 *(44.4)*	33 *(4.0)*	949 *(23.7)*	206 *(62.6)*	68 *(8.8)*	707 *(21.3)*	233 *(35.4)*
Promenade surrounding bandstand	317 *(19.9)*	1257 *(17.2)*	90 *(9.1)*	168 *(20.5)*	756 *(18.8)*	30 *(9.1)*	149 *(19.2)*	501 *(15.1)*	60 *(9.1)*
Other spaces	228 *(14.3)*	1527 *(20.8)*	252 *(25.5)*	122 *(14.9)*	714 *(17.8)*	37 *(11.3)*	106 *(13.7)*	813 *(24.5)*	215 *(32.6)*
Green spaces	949 *(59.5)*	2892 *(39.4)*	207*(21.0)*	497 *(60.6)*	1594 *(39.7)*	56 *(17.0)*	452 *(58.3)*	1298 *(39.1)*	151 *(22.9)*
Total	1595 *(100)*	7332 *(100)*	988 *(100)*	820 *(100)*	4013 *(100)*	329 *(100)*	775 *(100)*	3319 *(100)*	659 *(100)*

Numbers in parentheses are column percentages for the Chi Square Test

The sex-specific use of each target area showed similar trends. Green spaces had the highest use among both females (60.6%) and males (58.3%) in the < 18-years old category; this target area was also the most heavily used by adult females (39.7%) and males (39.1%). For seniors however, the area of highest use was the walking track for both females (62.6%) and males (35.4%).

#### Energy expenditure

Overall females had significantly lower mean EE scores than males (females = 0.078 kcal/kg/min, versus males = 0.080 kcal/kg/min, t = 3.11; *p* = 0.0018). Females had significantly lower mean EE than males for the walking track (females = 0.105 kcal/kg/min, versus males = 0.113 kcal/kg/min, t = − 8.92; *p* < 0.0001) and green spaces (females = 0.065 kcal/kg/min versus males = 0.069, t = − 2.31; *p* = 0.02). The mean difference in EE was not statistically significantly different between the sexes for the large promenade area surrounding the bandstand and the other areas (inclusive of small promenade areas, walking paths and areas with fixed concrete equipment).

The ANOVA results indicated that there were significant differences among the mean EE for the four target area types (see Table 3). The walking track had the highest mean EE, followed by the promenade surrounding the bandstand, with the green spaces having the least mean EE (F = 1209.20, p < 0.0001). Table 3 also reveals that the sex-specific mean EE by target area type showed a similar pattern with the mean EE highest on the walking track for females, (F = 581.32, p < 0.0001) and males (F = 628.69, p < 0.0001) respectively.

**Table 3:** Sex-specific mean Energy Expenditure (EE) by Target Area

Target Area	Total F = 1209.20, *p* < 0.0001			Females F = 581.32, *p* < 0.0001			Males F = 628.69, *p* < 0.0001		
	N	Mean EE	SD	N	Mean EE	SD	N	Mean EE	SD
Walking track	2195	0.109	0.021	1189	0.105	0.019	1006	0.113	0.023
Promenade surrounding bandstand	1694	0.077	0.033	969	0.076	0.034	725	0.077	0.033
Other spaces	2000	0.074	0.027	870	0.074	0.026	1130	0.074	0.028
Green spaces	4056	0.066	0.028	2151	0.065	0.027	1905	0.067	0.028
Total	9945	0.079	0.032	5179	0.078	0.031	4766	0.080	0.033

*EE* Energy Expenditure, *SD* Standard Deviation

### Qualitative findings

These qualitative findings provide depth and insight into perspectives of the users of the park and gives participants a voice that grounds study findings in the participants’ experiences. Specifically, park users comment on purposes for the use of the park, types of activities they engage in, as well as their energy expenditure and that of other users.

Five in-depth interviews were conducted with individuals in each activity level: two adult females (one sedentary, one vigorous), one adult male (vigorous) and two senior males (one vigorous and one walking).

#### Uses of Emancipation Park

##### Physical activity

Individuals used the park for walking, jogging, stretching and other movement activity as stated by a male participant, *“I can’t jog anymore so I do walk and after I try, I do some exercise.” G*roup-based physical activity also occurs as one park user recalled, *“I think I came here one evening and saw persons doing Taekwondo or something looking like that.”* Another observed *“a dancing aerobics class”* The PA included both adults and children *“On a very active day youths, young adults, middle-aged and young old of both sexes”.*

#### Relaxation and socialization

The park is used by people relaxing, being surrounded by nature even while in the middle of a commercial district. When asked the main reason for using the park one participant stated, *“the number one reason is for relaxation underneath the cool atmosphere.”* People also come for stress-relief provided by being in the physical space. For example, a participant observed being sedentary said, *“I love coming here, see the different persons, view whatever there is, relax you know, after a hard day’s work.”* Another participant claimed, *“people use it for a picnic area.”* Time spent in the park provides opportunities to socialize and network whether persons come to exercise or relax. One user shared about her experience and that of her friends *“We also built … not only a camaraderie of friends but other people because you meet people here.”* This view is shared by other participants *“after a while you meet people and discuss just about any and anything”* and *“Occasionally you will have a jogger have a little conversation with you.”*

### Health benefits

A few reasons for doing PA in the park were health related. One user stated, *“I was told by my doctor that I had to do exercises, that I had to be physical. So it was a health concern”.* Another user explained why so many persons were using the park for exercise, *“I think the objective of everyone coming here is to basically get themself in a better physical condition. Due to the fact that there is a lot of people suffering from all kinds of ailments. … to minimize that and keep people from having frequent visits to the doctors.”* Preventing illness and more specifically minimizing the economic and social burden of illness was motivational as stated by one participant *“the social cost of not exercising is the development of these diseases … As well as the economic costs of doing this because these illnesses … can almost bankrupt you.”*

#### Attributes associated with park use

One popular reason given for the choice of Emancipation Park was its proximity to work or home. One park user explained *“It is also convenient because it is relatively near to where I live”* while another said "*… ninety percent of the persons I see in the park are persons that would appear to be coming from work.* Its central location was another motivation for the park’s use as shared by another participant *"I actually walk from Crossroads. It is easy walking if you are in the exercise mode, and walk back".*

The park is also located close to hotels making it easily accessible for guests who are largely business tourists. A number of participants noted that similar parks were needed in the communities where they resided *"they should consider making more of these available in different communities* and *"I think people would use it* (a park).. *if you have them strategically placed.*

Aesthetic appeal was highly valued as an attribute for park use “*Well to be honest with you it is the really the scenery that I love”*. The presence of trees and other nature features made Emancipation Park attractive to some users “*I think it has to do with everything. It incorporates the trees, the grass … I used to come and I just lie in the grass and I would start doing push-ups”*. One participant felt this aspect motivated her to exercise *“The whole atmosphere at the park motivates you to want to do exercise”.*

The conditions and maintenance of the park were identified positive attributes. Participants’ listed the following features: “… *the water fountain”; “A laid out track*...”*;* “*… The surface … seems to be well cushioned so it helps*, *it saves you the problem for your knee”; “I find the physical layout attractive*”; “*Even the bathroom facilities. Well kept”*.

Safety and security was a key attribute for use of Emancipation Park. Park users alluded to feeling safe at the venue because of the visible presence of security officers to maintain order “*You always have security here*”. Most personal safety concerns mentioned by participants were associated with the presence of undesirable users of parks *“where the park is located, it is not surrounded by communities where people are deviant...”*

#### Barriers to use of the park for PA

The most frequently given barrier to the park’s use for PA however was overcrowding “*… it is too crowded … and if they get bumped you know...you can trip it’s just unpleasant.”* another stated *“at times it can get a little bit too crowded*” The overcrowding seems to drive people away from the park as this participant explained *“a lot of people complain to me that it is too crowded and as a consequence they go elsewhere”.*

A hot temperature was a barrier to day time use. For example, one participant commented *“I don’t really come to the park until in the evening after it is cooler”.*

## Discussion

To our knowledge this is the first reported systematic direct observation of park usage in a public recreational park in Jamaica. Our study revealed that SOPARC was a feasible and reliable instrument for assessing park users and associated contextual variables in keeping with findings from more developed countries [8, 20, 25]. The park was used mostly by females, in the evenings and by persons 18–64 years old. Females had significantly lower mean EE than males. The areas with highest use and highest levels of MVPA were the circumferential track, the promenade areas surrounding the bandstand areas and the large grassed open spaces at the south of the park. The interview data supported the direct observations of the park being a space that motivated engagement in PA. It also highlighted the importance of social environments for park use.

The application of this mixed methods approach provided a rich description of the activities and settings and their interaction within Emancipation Park. The findings from this study highlight that while the physical attributes of the park appear to encourage PA (e.g., walking path, perimeter fence, and presence of security officers); the relationship between context and behaviour was less than straight-forward. Thus, the physical and social characteristics as well as the location of the park are potentially important influences on park use for PA.

### Sex differences in park usage

Significantly more females than males visited the park. This is contrary to findings in a number of other observational studies on park user characteristics in the USA [8, 9, 14, 20] but similar to the findings by Veitch et al. in metropolitan parks in Australia [23]. Additional studies are needed to explore whether environmental attributes that positively influence park use (for e.g. aesthetics, security, paved trails, planned activities and proximity) [32–34] may be having a greater influence on females in this context or whether there are sex disparities in the composition of the workforce in the surrounding business district.

### Physical activity levels

Our findings that about 51% of all persons were engaged in sedentary behaviours are similar to other observation studies. For example, Floyd et al. [15] found a similar percentage among users of 10 parks in Tampa Florida. However even higher percentages of sedentary activities have been recorded in parks in other areas of the USA [8, 9, 15, 20]. The qualitative findings provide further insight into the multi-purpose appeal of the park including for sedentary purposes (recreation, family gatherings, relaxation, etc.) and indicate that some users have little interest in PA. However, this important attraction may provide an opportunity for policy makers to motivate users to engage in PA [8].

### Park location

The finding of greater use of the park by adults 18–64 years old and in the evenings, was expected given the location in the heart of a central business district. Many persons leave home early and return late and thus use of the park just before or after work may be quite convenient. It is possible that younger persons and seniors use parks and other open public spaces closer to their school and residential communities for PA. However, review of literature reveals an inconsistency in the associations of proximity and park use for PA. For example, Cohen et al. [8] used SOPARC and conducted interviews among park users in 8 public parks within the Los Angeles area and residents living within 2 miles of each park. They found that residential proximity was strongly associated with park use. However, Kaczynski et al. [35] in Canada, found that park proximity was not a significant predictor of park use for PA but park facilities were.

### Energy expenditure

The EE values obtained are semi-quantitative and are a weighted population metric to assess exercise intensity. Its usefulness resides in being a useful internal measure of exercise intensity by demographic characteristics and site. In keeping with other studies males were more engaged in more vigorous PA and overall had higher levels of mean EE [8–10, 15]. Given the burden of obesity among females and the greater use of this park by females, policymakers should consider introducing interventions in this and other public parks to increase the PA level among females. For example, in Brazil, Parra et al. [25] found that parks offering free supervised PA classes increased the PA level among females in parks versus those which did not.

### Park attributes and physical activity

The target area used most frequently was the circumferential walking track. Our observation suggests that including and maintaining a similarly surfaced walking/running track in similar public spaces will likely increase population PALs and mean EE. Our finding is similar to that of other studies. Reed et al. [14] used SOPARC and reported high use of paved trails compared to other activity settings for both males and females in 25 community parks in located in the USA. Besenyi et al. [21] assessed differences in age and reported that among seniors and adults using parks in Missouri USA, the highest EE was observed on paved trails compared to other park areas. Similar findings regarding positive associations of paved trails with park usage for PA, particularly among females have also been reported in other parts of the USA [35]. Based on the park rules, the paved circumferential path was only used for walking and running and was often overcrowded in the evenings. It is possible that there may have been increased use if the walking path was widened. Paved trails in other parks have been used for cycling as well and allowing this activity might have seen even higher PA levels for both sexes and among the younger age group. Aerobic type activities have also been associated with increased park use, especially among females [11]. This is the most likely reason why the promenade around the bandstand, which was observed to be used for aerobic activities, was amongst the areas with the high mean EE. Urban planners and policymakers in health should keep in mind the health benefits of paved paths and large promenade areas for fitness activities when developing public parks [36].

From an urban and social planning perspective, attributes of a park appear to be as important as its location in influencing usage. The qualitative findings suggest that park aesthetics, maintenance, amenities and safety/security have the potential to encourage use for PA. Emancipation Park, is the only place in Jamaica that has these combined attributes, with persons expressing a desire for similar parks in other communities. Policy makers can capitalize on these qualities to motivate and support users to expend higher levels of PA by providing structured activities within parks, and the other attributes for e.g. aesthetics, security, facilities etc. There is however some conflict in the literature regarding organized park activities. A study by Cohen et al. [37] showed that these types of initiatives (sporting events or exercise classes) tend to enhance park usage, but not necessarily PA (e.g., park users might be spectators). This contrasts with a study by Parra et al. among older adults in Bogotá Columbia [38] where cost-free, supervised PA classes were offered which found that, compared to people in parks without these classes park users were more likely to be seen engaging in moderate-to-vigorous (64% vs 49%) and vigorous (25% vs 10%) PA [25].

### Study limitations

There may be a few limitations to this study. Firstly, Inter-observer agreement (IOA) scores were not formally calculated during training and may have affected reliability assessment of gender-specific age and activity levels during the data collection period. Secondly, SOPARC uses momentary time sampling and so the duration of the users’ PA was not assessed. Third, data collection was limited to only one park and for only 16 days. The pre-determined observation periods may not have captured periods of increased use outside of those timeframes and secular variations. For example, we were only able to capture 1 aerobic session based on the sampling methodology. We also observed that many of these aerobic sessions started just after completion of the evening observation period. Future studies could expand the periods of observation to late evenings and explore whether many of the women observed sitting in the evening period may have been waiting for the start of such activities thereafter. Fourth, Emancipation Park is unique among recreational parks within Jamaica. There is greater security presence and a set of rules that limit the types of PA (for e.g. group sports such as soccer and sprinting on the circumferential track are not allowed). It is possible that park use and PA level may have differed by socioeconomic status (SES) as in other studies [15]. Future studies should examine whether park use and PA level are associated with these conditions and vary with SES. Fifthly, it is possible that the 5 five in-depth interviews did not allow sufficient insight into the motivations and barriers that may have influenced the observed park use. Additional surveys on park use and qualitative studies may provide more useful insights for increasing the use of the park for PA.

## Conclusions

This is the first study to describe the usage of a public park for PA in Jamaica and the Caribbean region. The study elicited aspects of park use for PA in a major urban park in Jamaica from different vantage points by using direct systematic observation augmented with a qualitative approach. It revealed important differential park use for PA by sex, age group and EE and provided insights into factors that motivate and hinder park usage for PA. The information from this study can be used by policymakers in Jamaica to inform PA intervention geared at addressing the high levels of obesity, particularly among females in Jamaica. Its design and features are positive attributes which can be used for future advocacy of well-designed public parks to promote increased usage and levels of MVPA by users. The baseline data provided can also be used for comparison with future studies in other parks across the island and in small island developing countries.

## Additional file

Additional file 1: Use of Emancipation Park for physical activity: Topic Guide – In-Depth Interview (PDF 82 kb)

## Data Availability

The datasets generated or analysed during this study are not publicly available as we are still using the data for other analyses. However, once the analyses are complete, we are willing to share the data on reasonable request.

## Abbreviations

ANOVA: Analysis of Variance
CARICOM: Caribbean Community
CI: Confidence Interval
EE: Energy expenditure
ICC: Intraclass correlation coefficient
IOA: Inter-observer agreement
JHLS: Jamaica Health and Lifestyle Survey
JYRRS: Jamaica Youth Risk and Resiliency Survey
MVPA: Moderate and Vigorous Physical Activity
NCDs: Non- Communicable Diseases
PA: Physical activity
S: Sedentary
SD: Standard Deviation
SES: Socioeconomic status
SOPARC: System for Observing Play and Recreation in Communities
TA: Target area
USA: United States of America
V: Vigorous
W: Walking

## References

[1] Abdulkadri A, Cunningham-Myrie C, Forrester T (2009). Economic burden of diabetes and hypertension in CARICOM states. Soc Econ Stud.

[2] Wilks R, Bennett F, Forrester T, McFarlane-Anderson N (1998). Chronic diseases: the new epidemic. West Indian Med J.

[3] Wilks R, Younger N, Tulloch-Reid M, McFarlane S, Francis D (2008). Jamaica health and lifestyle survey 2007–8.

[4] Francis DK, Van den Broeck J, Younger N, McFarlane S, Rudder K, Gordon-Strachan G (2009). Fast-food and sweetened beverage consumption: association with overweight and high waist circumference in adolescents. Public Health Nutr.

[5] Declaration of Port-Of-Spain: Uniting to Stop the Epidemic of Chronic NCDs: CARICOM. 2007. https://caricom.org/media-center/communications/statements-from-caricom-meetings/declaration-of-port-of-spain-uniting-to-stop-the-epidemic-of-chronic-ncds. Accessed 1 Apr 2018.

[6] Ministry of Health Jamaica (2013). National Strategic and Action Plan for the Prevention and Control of Non-communicable Diseases (NCDs) in Jamaica 2013- 2018.

[7] Jamaica Moves. Ministry of Health, Jamaica. https://www.jamaicamoves.com/. Accessed 21 Mar 2018.

[8] Cohen DA, McKenzie TL, Sehgal A, Williamson S, Golinelli D, Lurie N (2007). Contribution of public parks to physical activity. Am J Public Health.

[9] Chung-Do JJ, Davis E, Lee S, Jokura Y, Choy L, Maddock JE (2011). An observational study of physical activity in parks in Asian and Pacific islander communities in urban Honolulu, Hawaii, 2009. Prev Chronic Dis.

[10] Child ST, McKenzie TL, Arredondo EM, Elder JP, Martinez SM, Ayala GX (2014). Associations between park facilities, user demographics, and physical activity levels at San Diego County parks. J Park Recreat Admi.

[11] Tester J, Baker R (2009). Making the playfields even: evaluating the impact of an environmental intervention on park use and physical activity. Prev Med.

[12] McCormack GR, Giles-Corti B, Bulsara M, Pikora TJ (2006). Correlates of distances traveled to use recreational facilities for physical activity behaviors. Int J Behav Nutr Phys Act.

[13] Rung AL, Mowen AJ, Broyles ST, Gustat J (2011). The role of park conditions and features on park visitation and physical activity. J Phys Act Health.

[14] Reed JA, Arant C, Wells P, Stevens K, Hagen S, Harring H (2008). A descriptive examination of the most frequently used activity settings in 25 community parks using direct observation. J Phys Act Health.

[15] Floyd MF, Spengler JO, Maddock JE, Gobster PH, Suau LJ (2008). Park-based physical activity in diverse communities of two US cities: an observational study. Am J Prev Med.

[16] Bedimo-Rung AL, Mowen AJ, Cohen DA (2005). The significance of parks to physical activity and public health: a conceptual model. Am J Prev Med.

[17] Broyles ST, Mowen AJ, Theall KP, Gustat J, Rung AL (2011). Integrating social capital into a park-use and active-living framework. Am J Prev Med.

[18] Cunningham-Myrie CA, Theall KP, Younger NO, Mabile EA, Tulloch-Reid MK, Francis DK (2015). Associations between neighborhood effects and physical activity, obesity, and diabetes: the Jamaica health and lifestyle survey 2008. J Clin Epidemiol.

[19] Emancipation Park, Jamaica. Government of Jamaica. http://www.emancipationpark.org.jm/. Accessed 21 Mar 2018.

[20] McKenzie TL, Cohen DA, Sehgal A, Williamson S, Golinelli D (2006). System for observing play and recreation in communities (SOPARC): reliability and feasibility measures. J Phys Act Health.

[21] Besenyi GM, Kaczynski AT, Stanis SAW, Vaughan KB (2013). Demographic variations in observed energy expenditure across park activity areas. Prev Med.

[22] Floyd MF, Bocarro JN, Smith WR, Baran PK, Moore RC, Cosco NG (2011). Park-based physical activity among children and adolescents. Am J Prev Med.

[23] Veitch J, Carver A, Abbott G, Giles-Corti B, Timperio A, Salmon J (2015). How active are people in metropolitan parks? An observational study of park visitation in Australia. BMC Public Health.

[24] Van Dyck D, Sallis JF, Cardon G, Deforche B, Adams MA, Geremia C (2013). Associations of neighborhood characteristics with active park use: an observational study in two cities in the USA and Belgium. Int J Health Geogr.

[25] Parra DC, McKenzie TL, Ribeiro IC, Hino AAF, Dreisinger M, Coniglio K (2010). Assessing physical activity in public parks in Brazil using systematic observation. Am J Public Health.

[26] Cohen DA, Setodji C, Evenson KR, Ward P, Lapham S, Hillier A (2011). How much observation is enough? Refining the administration of SOPARC. J Phys Act Health.

[27] Creswell JW, Creswell JD (2018). Research design : qualitative, quantitative, and mixed methods approaches. Fifth edition.

[28] McKenzie T, Cohen D (2006). SOPARC (System for observing play and recreation in communities) description and procedures manual.

[29] Charmaz K. Qualitative interviewing and grounded theory analysis. In: Gubrium J, Holstein J, (Eds.). Handbook of interview research: Context and method. Thousand Oaks: SAGE Publications; 2001. pp. 675–94.

[30] Glaser BG (1965). The constant comparative method of qualitative analysis. Soc Probl.

[31] Flick U (2009). An introduction to qualitative research.

[32] McCormack GR, Rock M, Toohey AM, Hignell D (2010). Characteristics of urban parks associated with park use and physical activity: a review of qualitative research. Health Place..

[33] Giles-Corti B, Broomhall MH, Knuiman M, Collins C, Douglas K, Ng K (2005). Increasing walking: how important is distance to, attractiveness, and size of public open space?. Am J Prev Med.

[34] Kaczynski AT, Potwarka LR, Smale BJ, Havitz ME (2009). Association of parkland proximity with neighborhood and park-based physical activity: variations by gender and age. Leis Sci.

[35] Kaczynski AT, Potwarka LR, Saelens BE (2008). Association of park size, distance, and features with physical activity in neighborhood parks. Am J Public Health.

[36] Buchner DM, Gobster PH (2007). Promoting active visits to parks: models and strategies for transdisciplinary collaboration. J Phys Act Health.

[37] Cohen DA, Marsh T, Williamson S, Derose KP, Martinez H, Setodji C (2010). Parks and physical activity: why are some parks used more than others?. Prev Med.

[38] Parra DC, Gomez LF, Fleischer NL, David Pinzon J (2010). Built environment characteristics and perceived active park use among older adults: results from a multilevel study in Bogota. Health Place.

