# Supplementary material for: Use of a public park for physical activity in the Caribbean: evidence from a mixed methods study in Jamaica
Source: BMC Public Health. 2019 Jul 8;19:894. doi: 10.1186/s12889-019-7247-6 (PMC6615202; doi:10.1186/s12889-019-7247-6)
Supplement: Supplementary file 1 — Use of Emancipation Park for physical activity: Topic Guide – In-Depth Interview (PDF 82 kb) [file 12889_2019_7247_MOESM1_ESM.pdf]

Use of Emancipation Park for physical activity: Topic Guide – In-Depth Interview

[Written informed consent process completed]

Research Question: How and why do persons use Emancipation Park for physical activity?

Guiding Questions:

Proposed Time	Phase & Activities	Prompts / Probes
Three (3) minutes	***Introduction & Housekeeping*** (including consent):<br>• Approach participant.<br>• Introduce myself.<br>• Allow participant to introduce him/herself.<br>• Explain purpose of interview and clarify any related questions.	
Two (2) minutes	***Rapport Building:***<br>• Negotiate tape recording<br>o Participant has valuable information<br>o Difficult to document fully while speaking, hence request<br>o Personal identifying mark will be removed; tape will be destroyed after transcription<br>o Information provided can help guide future activities for health improvement in public parks<br>• Estimated interview time: twenty (20) minutes. May vary based on how much participant speaks.	
One (1) minute	***Transition to In-Depth Probing Phase***<br>OK. Let us now talk about the use of public spaces like parks.	
Three (3) minutes	***Question 1***: Why do you visit Emancipation Park?	1. How did you come to be aware of it?
Two (2) minutes	***Question 2***: Where do you come from to visit/use the Park?	1. Home? Work? School ? Other place or multiple places

Five (5) minutes	***Question 3***: Explain what you understand is meant by physical activity (PA)	2. What types of PA do you like to do?
Five (5) minutes	***Question 5***: How should persons be encouraged to use the Park for PA ?	1. Should there be different activities for different times of day?<br>2. What activities?<br>3. How often?<br>4. How long each time?
Three (3) minutes	***Question 6:*** Why would persons not want to use the Park for PA ?	
Two (2) minutes	***Closure***:<br>• Signal end of interview<br>• Offer to summarize for clarity and agreement<br>• Summarize and check for agreement<br>• Thank participant.	
